# Phase locked neural activity in the human brainstem predicts preference for musical consonance

**DOI:** 10.1016/j.neuropsychologia.2014.03.011

**Published:** 2014-05

**Authors:** Oliver Bones, Kathryn Hopkins, Ananthanarayan Krishnan, Christopher J. Plack

**Affiliations:** a School of Psychological Sciences, The University of Manchester, Manchester M13 9PL, UK; b Department of Speech, Language, and Hearing Sciences, Purdue University, West Lafayette, IN 47907, USA

**Keywords:** Musical consonance, Individual differences, Auditory brainstem, Frequency following response, Pitch, Harmonicity

## Abstract

When musical notes are combined to make a chord, the closeness of fit of the combined spectrum to a single harmonic series (the ‘harmonicity’ of the chord) predicts the perceived consonance (how pleasant and stable the chord sounds; McDermott, Lehr, & Oxenham, 2010). The distinction between consonance and dissonance is central to Western musical form. Harmonicity is represented in the temporal firing patterns of populations of brainstem neurons. The current study investigates the role of brainstem temporal coding of harmonicity in the perception of consonance. Individual preference for consonant over dissonant chords was measured using a rating scale for pairs of simultaneous notes. In order to investigate the effects of cochlear interactions, notes were presented in two ways: both notes to both ears or each note to different ears. The electrophysiological frequency following response (FFR), reflecting sustained neural activity in the brainstem synchronised to the stimulus, was also measured. When both notes were presented to both ears the perceptual distinction between consonant and dissonant chords was stronger than when the notes were presented to different ears. In the condition in which both notes were presented to the both ears additional low-frequency components, corresponding to difference tones resulting from nonlinear cochlear processing, were observable in the FFR effectively enhancing the neural harmonicity of consonant chords but not dissonant chords. Suppressing the cochlear envelope component of the FFR also suppressed the additional frequency components. This suggests that, in the case of consonant chords, difference tones generated by interactions between notes in the cochlea enhance the perception of consonance. Furthermore, individuals with a greater distinction between consonant and dissonant chords in the FFR to individual harmonics had a stronger preference for consonant over dissonant chords. Overall, the results provide compelling evidence for the role of neural temporal coding in the perception of consonance, and suggest that the representation of harmonicity in phase locked neural firing drives the perception of consonance.

## Highlights

• Harmonic structure of musical chords is encoded by phase locking in the brainstem.
• Individual preference for consonance can be predicted by neural coding.
• The neural coding of the harmonicity of a musical chord predicts how pleasant it is.
• Monaural interactions generate neurally coded harmonics.
• Generated harmonics enhance neural harmonicity and pleasantness of consonant chords.

## 1 Introduction

When two musical notes are played together, such as when two piano keys are pressed simultaneously, the result is a type of chord called a ‘dyad.’ For example, the consonant dyad the Perfect 5th is created by combining a lower note with a note that is seven keys higher on a piano. The term ‘consonance’ is used to describe combinations of notes which result in a pleasing perception of resolution and stability. In contrast ‘dissonance’ is used to describe combinations which produce an unpleasant perception of being unresolved and unstable. The fact that consonant combinations are deemed preferable to dissonant combinations (McDermott et al. 2010; Plomp & Levelt, 1965) contributes to a sense of musical key (Rameau, 1971). Music is a universal human phenomenon (e.g. McDermott & Hauser, 2005); an understanding of the auditory processes involved in listening to music may reveal which musical parameters are determined by innate factors, and uncover the mechanisms which are defective in those individuals with an impaired perception of the distinction between consonance and dissonance, and therefore an impaired enjoyment of music (e.g. those with sensorineural hearing loss; Tufts, Molis, & Leek, 2005).

An individual note produced by a musical instrument is an example of a complex tone. The spectrum of a complex tone contains a number of components called ‘harmonics’ with frequencies at integer multiples of the fundamental frequency (*F*0). For example, the musical note ‘C’ has an *F*0 of 130.81 Hz and harmonics at 261.62, 392.43, 523.24, 654.05 Hz etc. The *F*0 is also the frequency of the overall repetition rate of the waveform. When a complex tone enters the cochlea the low-numbered harmonics are separated out (‘resolved’), with each harmonic exciting a distinct place on the basilar membrane. Information about the frequency of resolved components is encoded in the auditory nerve by the tendency of auditory nerve fibres to synchronise their firing to the *temporal fine structure* (TFS) of the basilar membrane vibration (‘phase locking’; Brugge, Anderson, Hind, & Rose, 1969; Cariani & Delgutte, 1996; Rose, Hind, Anderson, & Brugge, 1971) so that the corresponding period of each resolved harmonic is represented in the inter-spike intervals (ISI) of the fibres innervating a place on the basilar membrane corresponding to that frequency. In addition, neurons will also tend to phase lock to the overall amplitude of the vibration over time (the *temporal envelope*) produced by the interactions of harmonics on the basilar membrane (Cariani & Delgutte, 1996; Hall, 1979). The dominant modulation rate of these interactions is equal to the frequency spacing of the harmonics (the *F*0 in the case of harmonic complex tones).

**Fig. 1:**
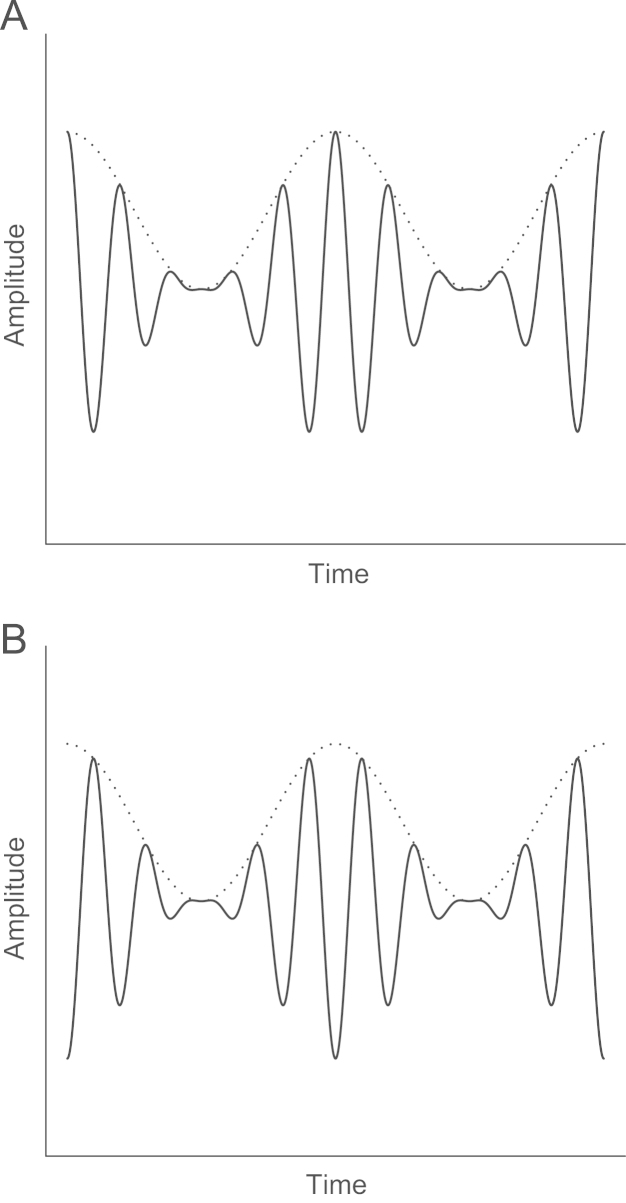
A section of a modulated waveform with its original polarity (A) and polarity inverted 180 degrees (B). Phase locking to the TFS (solid line) of each stimulus will be in opposite phase. Phase locking to the envelope (dotted line) of each stimulus will be in the same phase.

The scalp-recorded electrophysiological frequency following response (FFR) is a measure of neural phase locking in the brainstem (Moushegian, Rupert, & Stillman, 1973). The FFR has been widely used to explore the relation between temporal coding at this stage of the auditory pathway and pitch perception (Anderson, Parbery-Clark, White-Schwoch, & Kraus, 2012; Anderson, White-Schwoch, Parbery-Clark, & Kraus, 2013; Bidelman, Gandour, & Krishnan, 2011a, 2011b; Bidelman & Heinz, 2011; Bidelman & Krishnan, 2009; Carcagno & Plack, 2011; Clinard, Tremblay, & Krishnan, 2010; Gockel, Carlyon, Mehta, & Plack, 2011; Krishnan, Bidelman, & Gandour, 2010; Krishnan, Bidelman, Smalt, Ananthakrishnan, & Gandour, 2012; Krishnan & Plack, 2011; Krishnan, Xu, Gandour, & Cariani, 2005; Wong, Skoe, Russo, Dees, & Kraus, 2007). The FFR to a complex tone contains phase locked activity to both the cochlear envelope and the TFS. However, Goblick and Pfeiffer (1969) developed a method for selectively enhancing the FFR to either of these components: the FFR is averaged separately across trials with the stimulus presented in the original onset polarity and across trials with the onset polarity inverted 180 degrees (see Fig. 1). Adding the two averages together has the effect of suppressing the FFR to the TFS whilst enhancing the response to the envelope; subtracting the averages has the opposite effect of suppressing the response to the envelope whilst enhancing the response to the TFS. This technique was employed in the present study to explore the separate contributions made by the neural representation of the temporal envelope and of the TFS of musical dyads in the perception of musical consonance.

Explanations for the perception of consonance have been debated for many hundreds of years. Observing the behaviour of plucked strings, the ancient Greeks noted that when two notes are combined, simple vibration ratios produce consonant sounds whilst complex ratios produce dissonant sounds. For example, the frequency ratio of the highly consonant Perfect 5th interval is approximately 3:2, whereas the ratio of the dissonant Tritone interval is approximately √2:1. A consequence of complex frequency ratios is that the combined frequency spectrum frequently contains harmonics which are closely spaced on the basilar membrane. Many modern accounts of consonance and dissonance have been informed by Helmholtz׳s (1954) theory of auditory ‘beating’ (e.g. Plomp, 1964; Plomp & Levelt, 1965; Rasch & Plomp, 1999). This theory states that the perception of dissonance occurs when harmonics which are closely spaced on the basilar membrane interact with one another, causing amplitude modulation (beating) and a sensation of ‘roughness’. The auditory beating theory suggests that consonance is the perception that occurs in the absence of roughness. It is clear however that this is an insufficient explanation for musical consonance. At low *F*0s even consonant frequency ratios result in dyads with low frequency components that are closely spaced enough for beating to occur (Terhardt, 1974).

The magnitude spectra of dyads with simple frequency ratios such as the Perfect 5th interval closely resemble a single harmonic series and therefore a single musical note. McDermott et al. (2010) demonstrated that the perception of consonance is likely to be driven by the closeness of fit of the combined harmonics from the two notes to a single harmonic series (the ‘harmonicity’; see also Cousineau, McDermott, & Peretz, 2012). Individuals with a stronger preference for harmonicity in non-musical tones also had a stronger preference for consonant dyads over dissonant dyads. Individual ratings of the unpleasantness of beating on the other hand were not related to preference for consonance. Musical experience was found to significantly correlate with preference for both harmonicity and consonance, but not for absence of beating, further indicating the importance of harmonicity to music. The results of this study provide compelling evidence that it is harmonicity resulting from simple frequency ratios that drives the perception of consonance, rather than simply the absence of beating.

There are a number of reasons why harmonicity might be important to the perception of consonance. Mathematical models of sub-cortical ISI processing have been used to explain psychophysical phenomena such as frequency discrimination (Meddis & Hewitt, 1991a, 1991b; Meddis & O׳Mard, 1997) and the perception of consonance (Ebeling, 2008). In Ebeling׳s model it is the coincidence of neural firing when frequency components are harmonically related (and therefore have overlapping periods) that leads to the perception of consonance. In a bid to explain ‘virtual pitch’ (the pitch produced by a harmonic series consisting only of harmonics above a ‘missing’ fundamental or first harmonic) Terhardt (1974, 1979, 1984) proposed a harmonic template detection schema. In this schema pitch is determined by matching the combined frequency components of a sound to the best fitting harmonic series by finding the sub-harmonic (the low frequency component not present in the stimulus with a frequency of *f/n* where *n* is an integer) shared by the most harmonics present. Consonance is the perception of ‘tonal affinity’ when the combined spectra of two or more complexes have a strong ‘fundamental-tone relation’ (Terhardt, 1977). Similarly Stumpf (1890) conceived of consonance as the perceptual consequence of ‘tonal fusion’ i.e. the perception that occurs when two notes perceptually fuse into a single entity. One way in which two musical notes might fuse is by their harmonics closely resembling a single harmonic series with a single *F*0 and therefore a single pitch. A similar idea was held by 18th century German philosopher Rameau (1971) who believed that the individual notes of a consonant chord could be related to one another by a ‘fundamental bass note,’ i.e. a shared sub-harmonic. Models of consonance based on neuronal periodicity detection schemes (e.g. Ebeling, 2008) use information coded by the phase locking of neurons in the subcortical auditory pathway. Tramo, Cariani, Delgutte, and Braida (2001) observed that the dominant period in consonant stimuli (corresponding to a shared sub-harmonic of the individual notes) could be observed in the pooled ISI histograms of the auditory nerve fibres of cats. Previous FFR studies have found that musical interval has a significant effect on the salience of the period corresponding to the lower note of the dyad׳s *F*0 in phase locked brainstem activity, and that there is a strong correlation between the consonance of an interval and the average salience of this period (Bidelman & Krishnan, 2009, 2011). One aim of the present study was to test the hypothesis that the perception of consonance can be accounted for by the neural representation of harmonicity.

**Fig. 2:**
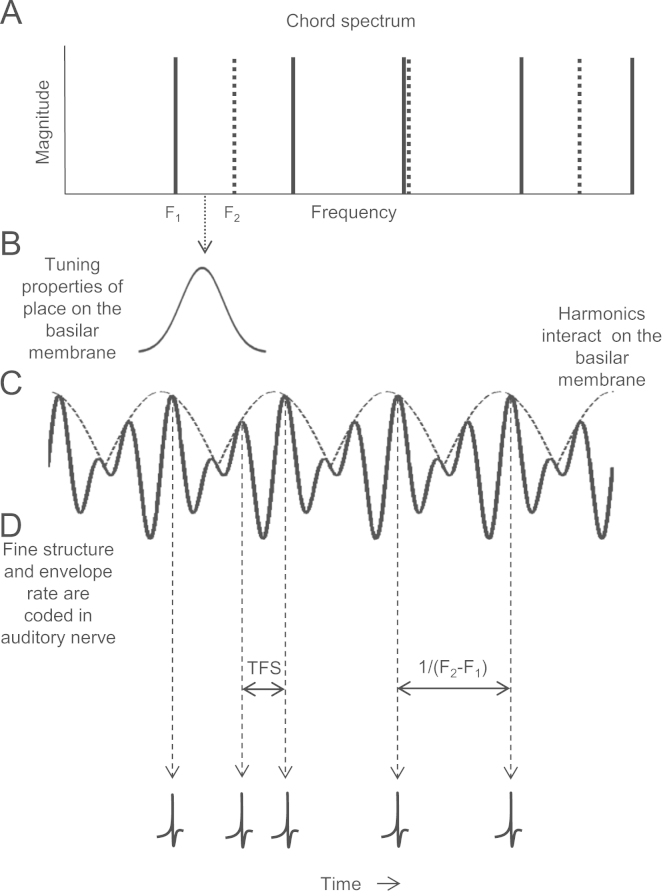
(A) The spectrum of a consonant musical dyad (the Perfect 5th). Solid lines indicate frequency components of the root note, dotted lines represent frequency components of the interval note. The combined spectrum contains harmonics which are regularly spaced and often separated by a harmonically relevant frequency difference. Where harmonics coincide (the third harmonic of the root note, and the second harmonic of the interval note) the harmonic from the interval note has been slightly off-set so that both are shown. Harmonics interact most strongly at a place on the basilar membrane with a characteristic frequency approximately in the middle of the two harmonics (indicated here by the downwards arrow). Although resolved from one another at their characteristic place on the basilar membrane, harmonics interact within the cochlear auditory filter with a characteristic frequency between the two harmonic frequencies (B). The resulting vibration pattern has a TFS (solid line) and an envelope (dotted line; C) Neurons will tend to phase lock to the envelope modulation rate (equal to the difference between the interacting frequency components; *F*_2_–*F*_1_) as well as to the TFS (D).

If consonance depends on the neural representation of harmonicity, it might be expected that perception would be dependent on the way the notes are presented to the two ears. When two notes of a consonant chord are presented to the same ear (or both notes to both ears; ‘diotically’), due to the regular spacing of the combined harmonics the interactions between the two notes on the basilar membrane produce temporal envelopes containing frequency components that are *harmonically related* to the components of the combined spectrum. For example, the *F*0s of the two notes of the C Perfect 5th dyad are 130.81 and 196.00 Hz. Therefore an envelope component of approximately 65 Hz will be present (see Fig. 2). The three frequencies are all approximately harmonics of a 65 Hz fundamental. Hence, phase locking to the envelope produced by harmonic interactions between the notes in a consonant chord may effectively reinforce the harmonicity of the neural representation of the combined spectrum. When musical notes enter different ears (‘dichotically’), cochlear interaction between *F*0s is not possible and therefore enhancement of harmonicity in this way cannot occur.

The present study was designed to test the following hypotheses. (1) Individual differences in preference for consonant dyads over dissonant dyads are related to individual differences in the relative strength of the temporally coded neural representation of the harmonicity of these dyads. (2) Presenting both notes to both ears (‘diotic’ presentation) results in a stronger perception of consonance compared with presenting notes to different ears (‘dichotic’ presentation), due to the contribution of temporal interactions between the harmonics of the two notes on the basilar membrane that reinforce the harmonic series. (3) Temporal interactions between the harmonics of the two notes in the diotic case lead to a stronger neural representation of the harmonic series compared with the dichotic case.

## 2 Materials and methods

### 2.1 Participants

Thirty-two young normal-hearing participants with no history of neurological disorders, speech or language difficulties, or tinnitus completed behavioural testing. Four were removed from the data set as outliers. These participants had consonance preference scores more than two standard deviations below the mean, due to inconsistent interval ratings across trials (the mean ratings for each interval were approximately equal). Of the remaining 28 participants (mean age, 22 years; range, 18–30 years; 18 females) 19 completed both behavioural testing and electrophysiological testing (mean age, 22 years; range, 18–27 years; eight females). Hearing ability was assessed using pure-tone audiometry. Hearing thresholds for all participants were 20 dB HL or better for frequencies ranging from 500 to 8000 Hz.

Participants completed a questionnaire on their experience playing musical instruments. They indicated how many hours per week they played their instruments and the number of years that this applied to, with the option of indicating different periods of practice. For example, they could specify that they played their instrument for 10 h per week between the ages of 10 and 14, and then for 2 h per week from the age of 14 until the age of 20. Musical experience was then estimated by calculating the total number of hours practice for each participant. Musical experience ranged from 0 h to 13000 h.

### 2.2 Behavioural testing

#### 2.2.1 Stimuli

**Fig. 3:**
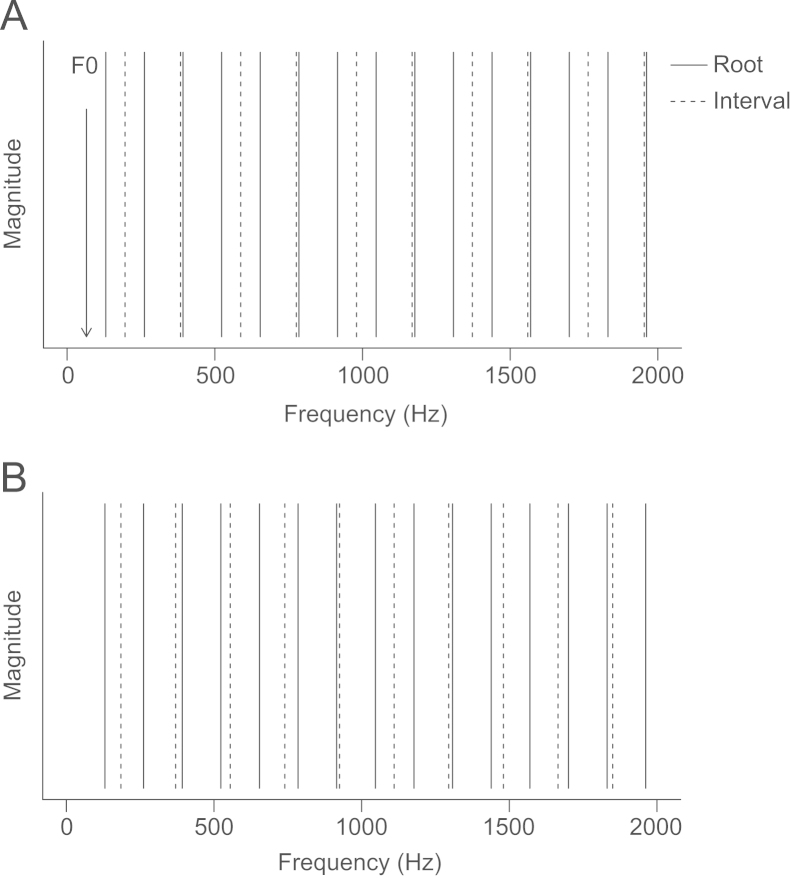
Schematic representation of a consonant interval, the Perfect 5th (A), and a dissonant interval, the Tritone (B). Solid lines indicate frequency components of the root note, dotted lines represent frequency components of the interval note. Where frequency components coincide the interval note harmonic has been displaced slightly so that both are shown. In the Perfect 5th the two sets of frequency components form a harmonic series of a ‘missing’ fundamental frequency, indicated by the downward arrow (A). In the Tritone the combined frequency components do not fit a harmonic series (B).

Stimuli were dyads (two note chords) made up of a lower note (the ‘root’ note) and a higher note (the ‘interval’ note, i.e. the note that defines the distance between the two notes and therefore the name of the dyad). Four root notes were used for the consonance preference testing, all taken from the equal-temperament scale: A (110.00 Hz); C (130.81 Hz); D♯ (155.56 Hz); and F# (185.00 Hz). These were each combined with six ‘interval notes’ to produce 24 dyads. Each interval note is defined by its ratio to the root note (see Table 1). The resulting complexes contain frequency components from two harmonic series. In the case of consonant intervals such as the Perfect 5th, the combined harmonics form a spectrum with frequency components which approximate a single harmonic series (see Fig. 3A). In the case of dissonant intervals such as the Tritone, the combined harmonics are irregularly spaced and there is no clear harmonicity (see Fig. 3B).

Each dyad was low-pass filtered at 2000 Hz. For each note the harmonics in the pass-band of the filter were of equal amplitude, set so that the overall level of each note was 80 dB SPL for dichotic presentation and 77 dB SPL for diotic presentation (to correct for the fact that two notes were presented to each ear in the diotic case). Each dyad was 2000 ms in duration, including 10 ms raised-cosine onset and offset ramps,

Each dyad was preceded by wide-band Gaussian noise with a 2 s duration including 10 ms raised-cosine onset and offset ramps. The noise was low-pass filtered at 2000 Hz. A 500 ms silence separated the wide-band noise and the dyad. The purpose of the noise was to break up the sequence of dyads in order to prevent melodic structure from influencing responses (McDermott et al., 2010). All stimuli were generated digitally at a sampling rate of 24,414 Hz with 32-bit resolution. Stimuli were delivered via a 24-bit E-MU 0202 USB audio device and Sennheiser HD 650 supra-aural headphones.

#### 2.2.2 Procedure

In order to investigate the effects of temporal interactions created by presenting two notes to the same ear, two conditions were tested: one in which both notes were presented to both ears (diotic condition), and one in which the root note was presented to the right ear, and the interval note was presented to the left ear (dichotic condition). Behavioural ratings and individual consonance preference were measured by following the methodology of McDermott et al. (2010). Participants were instructed to rate each dyad for pleasantness using a scale from −3 (very unpleasant) to +3 (very pleasant). Each run consisted of 48 stimuli (24 dyads, diotic and dichotic) presented in a random order. Responses from four runs were recorded, with all runs performed consecutively on the same day. Prior to the test, participants completed a practice run consisting of one of each interval in order to familiarise them with the procedure and the range of intervals used. Participants were seated in a sound-attenuating booth and responded via a keyboard and a computer display visible through a window in the booth.

#### 2.2.3 Analysis

Ratings for each interval were averaged across runs for each presentation condition (diotic and dichotic). In order to calculate consonance preference, averaged ratings were first *z-*scored for each individual in order to remove the influence of individual differences in the use of the scale (McDermott et al., 2010). Consonance preference was then calculated by subtracting each individual׳s average *z*-scored rating of the three theoretically most dissonant intervals (the Minor 2nd, the Tritone, and the Major 7th) from the average *z*-scored rating of the three most consonant intervals (the Perfect 4th, the Perfect 5th, and the Major 6th), as determined *a priori* by Western tonal music tradition (Rameau, 1971).

### 2.3 Electrophysiology

#### 2.3.1 Stimuli and recording procedure

Stimuli were a subset of the dyads used for the behavioural measures. The root note was C (130.81 Hz), taken from the equal-temperament scale, with the same six interval notes used in the consonance preference test (see Table 1). Each dyad was presented in a diotic condition and a dichotic condition, meaning that 12 dyads were presented to each participant in a randomised sequence. Each stimulus was 120 ms in duration, including 10 ms raised-cosine onset and offset ramps. Filtering and presentation level was the same as for the behavioural procedure. Each presentation window contained two stimuli separated by 120 ms silence. In order to use the method described by Goblick and Pfeiffer (1969) for enhancing the FFR to either the cochlear envelope or the TFS, the onset polarity of the second stimulus in the pair was inverted 180 degrees with respect to the onset polarity of the first stimulus (see Fig. 1). Presentations consisting of the two stimuli repeated at a rate of 2.09/s. FFR waveforms were averaged across 2000 presentations of each polarity.

Participants were seated in a comfortable reclining chair in a sound-attenuating booth and told that they could sleep. Stimuli were delivered via a TDT RP2.1 Enhanced Real Time Processor and HB7 Headphone Driver and Etymotic ER30 transducers. The length of the ER30 tubing connecting the transducers to the ear tips made it possible to position the transducers outside of the recording booth, therefore preventing stimulus artefacts from affecting the recording. This is a particular concern in FFR recordings because the electrophysiological response and the stimulus share the same frequencies. Recordings contaminated by the transduction of stimulus harmonics can easily be mistaken for neural activity phase locked to that frequency. Tubing was visually inspected for kinks before each session.

The FFR was recorded using TDT BioSig software with high-pass filtering at 30 Hz, low-pass filtering at 3000 Hz, and a notch filter at 50 Hz to remove mains electrical noise. A vertical electrode montage was used, with an active electrode at the high forehead hairline, a reference electrode at the seventh cervical vertebra, and a ground at Fpz (Bidelman & Krishnan, 2009, 2011; Krishnan & Plack, 2011; Krishnan et al. 2005). Impedances were maintained below 5 kΩ. As the BioSig software did not permit continuous recording, data was compiled online as 200 sub-averages of 10 responses (the smallest sized sub-average permitted by the equipment) to each stimulus polarity. Any sub-average of ten sweeps in which the peak amplitude exceeded +/- 30 μV at any time during the waveform was considered an artefact and removed offline. Responses were digitally high-pass filtered at 45 Hz offline in order to attenuate frequencies containing cortical responses further.

#### 2.3.2 Analysis

In order to determine the strength of harmonicity in the FFR, a measure of harmonic salience was derived in the following way. First the best fitting harmonic series was determined for the power spectrum of each stimulus. A fast-Fourier transform (FFT) was performed for each stimulus waveform. The power spectrum was then analysed by measuring the power inside of 4 Hz-wide bins placed at integer multiple frequencies of the *F*0. Harmonic salience was calculated as the ratio between the sum of power inside the bins and the sum of power outside of the bins for *F*0s ranging from 30 to 1000 Hz in 0.01 Hz step-sizes. 30 Hz is considered to be the lower limit of musical pitch (Pressnitzer, Patterson, & Krumbholz, 2001), whilst the upper limit was chosen so as to be well above the *F*0s of all of the notes used. Table 1 contains a summary of the *F*0s that resulted in the highest harmonic salience (taken to be the *F*0s of the best fitting harmonic series) for each stimulus. This information was then used to analyse the FFR. An FFT was performed for each FFR, and the harmonic sieve analysis described above performed with an *F*0 corresponding to the best fitting harmonic series to the stimulus. This salience measure was used to estimate the strength of harmonicity in the neural response. The routine was implemented in MATLAB using a script adapted from Bidelman and Krishnan (2009). The procedure described here is an adaptation of the ‘pitch salience’ measure described by the same authors, who analysed the autocorrelation function of the FFR to derive a measure of the strength of the periodicity corresponding to the root note *F*0. The analysis of the autocorrelation function of the FFR described by Bidelman and Krishnan (2009) results in a measure of the strength of the representation of a given period (1/*F*0) and its integer multiples (*n*/*F*0) in the FFR waveform. The present method differs from that used by Bidelman & Krishnan in two respects: (1) the analysis was performed on the power spectrum of the response; and (2) the best harmonic fit was found for each stimulus *a priori* as described above rather than using the root note *F*0 to determine the harmonic sieve. This method was used rather than the method employed by Bidelman and Krishnan (2009) since the purpose of the study was to explore the effects of the neural representation of harmonicity, and the harmonic series of the root note׳s *F*0 (or its sub-harmonics *n*/*F*0) did not always correspond to the best fitting harmonic series of the stimulus (see Table 1). In addition, performing analysis on the power spectrum in the current study allowed for the visualisation of the role of individual frequency components in supporting harmonicity.

In order to measure the strength of the representation of harmonicity in the FFR to consonant relative to dissonant intervals for each individual, a neural consonance index (NCI) was calculated using a method similar to that used to calculate behavioural consonance preference: each individual׳s average harmonic salience score for the three dissonant intervals was subtracted from their average harmonic salience score for the three most consonant intervals, determined *a priori* as in the behavioural analysis.

An aim of this study was to assess the separate contributions made by the neural representation of the cochlear envelope and TFS to the perception of consonance. In order to do this, analyses were performed on three FFR ‘types.’ First, the FFR containing both cochlear envelope and TFS components was analysed (FFR_RAW_) by performing the harmonicity analysis described above on the mean power spectrum of the direct polarity and inverted polarity FFR. Where FFR_RAW_ spectra are plotted they are averages from the direct and inverted polarity response i.e. the spectra upon which analyses were performed. In the interests of simplicity, rather than plot two waveforms (the responses to both polarity stimuli) where FFR_RAW_ waveforms are plotted they are responses to the direct polarity stimulus. Recording responses to a direct polarity and to an inverted polarity version of each stimulus allowed analyses to be performed on a second FFR type with the response to the envelope suppressed and TFS enhanced. The second FFR type was created by subtracting the FFR to the inverted stimulus polarity from the FFR to the direct stimulus polarity (FFR_SUB_). The inverted polarity stimulus contains harmonics that are in opposite phase to those in the direct polarity stimulus, but has an envelope that is in the same phase (see Fig. 1). By subtracting the responses to the two waveforms, the contribution of phase locking to the temporal envelope component is reduced and the contribution of phase locking to the TFS (phase locking to individual harmonics) is enhanced. Thirdly, by adding the FFR of the direct stimulus polarity and the inverted polarity (FFR_ADD_) phase locking to the envelope is enhanced and phase locking to TFS is suppressed (Goblick & Pfeiffer, 1969). An FFT was performed for each FFR type and harmonic salience calculated as described above. The NCI calculated from FFR_RAW,_ FFR_SUB_ and FFR_ADD_ is hereafter referred to as NCI_RAW,_ NCI_SUB_ and NCI_ADD_ respectively.

## 3 Results

### 3.1 Individual differences in consonance preference and the harmonicity of phase locking

**Fig. 4:**
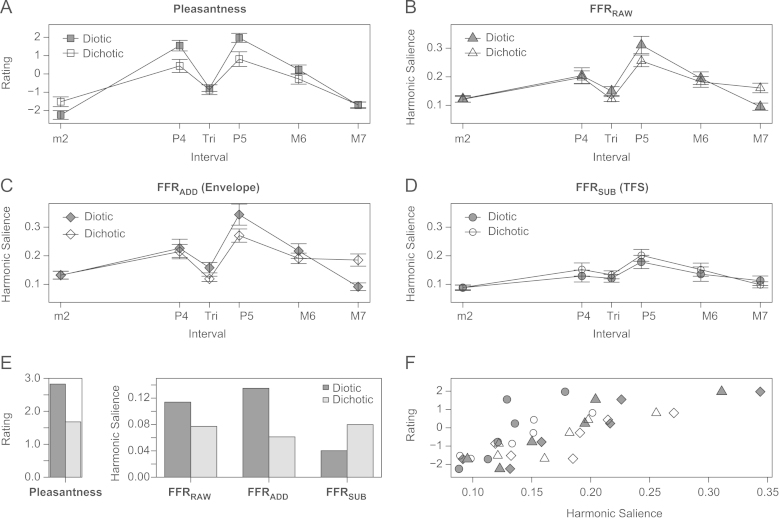
For all panels, filled symbols represent the diotic condition and empty symbols represent the dichotic condition. Error bars indicate 95% confidence intervals. Average pleasantness ratings plotted as a function of interval (A). Average harmonic salience as a function of interval for FFR_RAW_ (FFR to both envelope and TFS, B), FFR_ADD_ (FFR with contributions from the envelope enhanced and contributions from TFS suppressed, C) and FFR_SUB_ (FFR with contributions from the envelope suppressed and contributions from TFS enhanced, D). The summary data plot displays the difference between the average consonant and dissonant interval pleasantness rating and harmonic salience, for diotic and dichotic presentation (E). Average pleasantness ratings of each interval plotted as a function of harmonic salience. As for other panels, triangles represent FFR_RAW_, diamonds represent FFR_ADD_ and circles represent FFR_SUB_ (F).

Average dyad pleasantness ratings are displayed in Fig. 4A. The pattern of ratings for different intervals is consistent with previous studies involving normal-hearing listeners (Bidelman & Krishnan, 2009; McDermott et al., 2010): the Perfect 5th was rated most pleasant, and the Perfect 4th, the Major 6th, and the Tritone were rated progressively less pleasant. In the diotic condition the Minor 2nd is rated as least pleasant, whereas in the dichotic condition the Major 7th is rated as least pleasant. Note also the effect of presentation condition (diotic or dichotic). The effects of presentation condition in Fig. 4A–D are summarised in Fig. 4E and are discussed in more detail in Sections 3.2 and 3.3.

The average harmonic salience for each interval for FFR_RAW,_ FFR_SUB_ and FFR_ADD_ are plotted in Fig. 4B, C and D respectively. Note that the plots are similar to the plot of pleasantness ratings in Fig. 4A, with the consonant intervals resulting in higher harmonic salience than the dissonant intervals in each case. Overall, harmonic salience scores were lower for the FFR_SUB_ data than in the other two FFR types.

Interval pleasantness ratings are plotted as a function of harmonic salience in Fig. 4F. There was a strong correlation between average harmonic salience in the FFR and average behavioural pleasantness rating (paired by interval) for each presentation condition and FFR type (FFR_RAW_ diotic, *r*_s_(4)=0.94, *p*<0.01, dichotic, *r*_s_(4)=0.83, *p*=0.02; FFR_SUB_ diotic, *r*_s_(4)=0.94, *p*<0.01, dichotic, *r*_s_(4)=0.94, *p*=0.01, FFR_ADD_ diotic, *r*_s_(4)=0.94, *p*<0.01, dichotic *r*_s_(4)=0.77, *p*=0.04). Due to data being non-normally distributed, all reported correlation coefficients are Spearman׳s Rho.

**Fig. 5:**
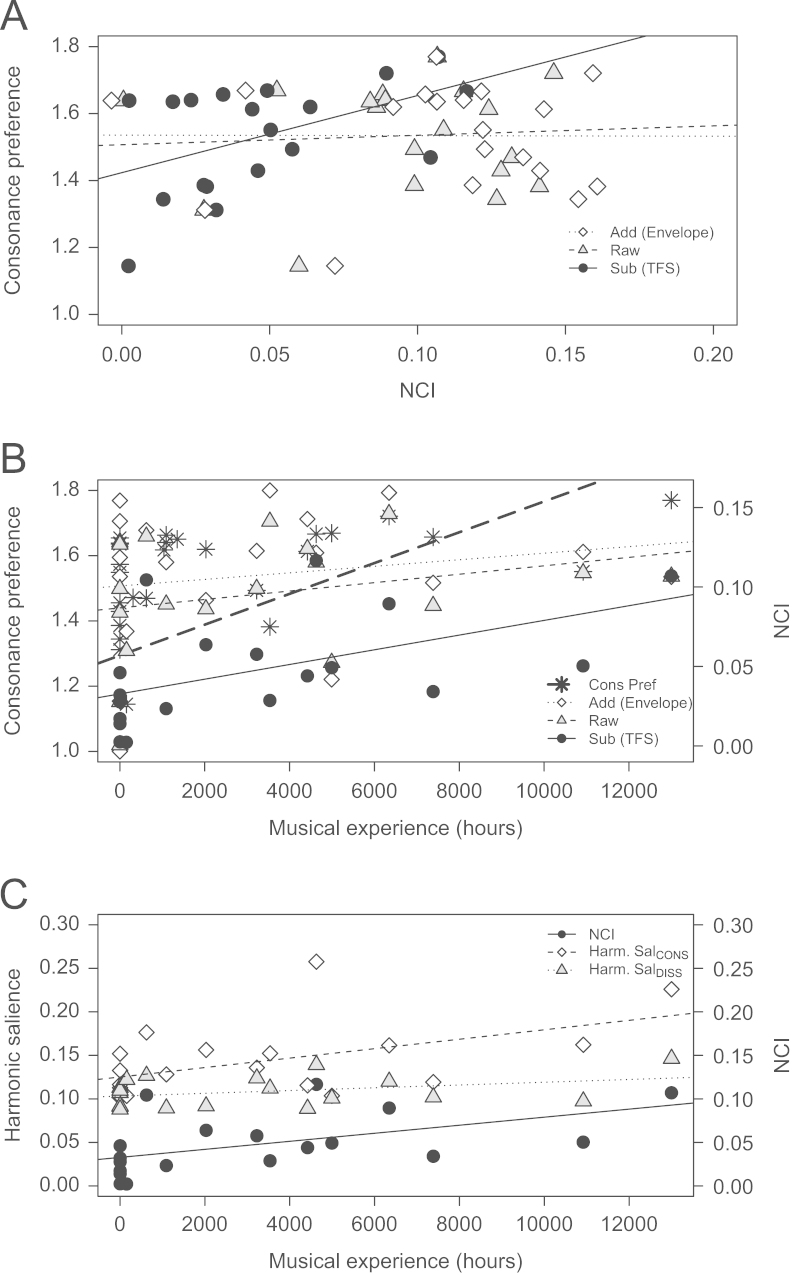
Individual consonance preference scores plotted as a function of NCI calculated from each FFR type (A). Consonance preference (left axis) and NCI calculated from each FFR type (right axis) as a function of musical experience (B). Harmonic salience of the FFR_SUB_ to consonant (Harm. Sal_CONS_) and dissonant dyads Harm. Sal_DISS_; (left axis), and NCI_SUB_ (right axis) as a function of musical experience (C).

Consonance preference and NCI scores were averaged across diotic and dichotic conditions for each participant and tested for correlation (see Fig. 5A). In Bonferroni corrected one-tailed tests consonance preference was found to significantly correlate with NCI_SUB_ (*r*_s(17)_=0.49, *p*=0.02), but not NCI_RAW_ (*r*_s(17)_=−0.04, *p*=0.44) nor NCI_ADD_ (*r*_s(17)_=−0.16, *p*=0.26). NCI_SUB_ represents more salient harmonicity in the phase locked neural firing to TFS of consonant dyads relative to dissonant dyads. To test this correlation further individual consonance preference scores were again correlated with NCI_RAW_, this time partialing out NCI_ADD_ and thus the confounding effect of individual variance in temporal envelope coding. The correlation coefficient rose from −0.04 to 0.36, consistent with there being a relation between NCI_RAW_ and consonance preference, although the correlation was marginally non-significant (*r*_s(16)_=0.36, *p*=0.07).

As expected, musical experience was significantly correlated with consonance preference (*r*_s(26)_=0.64, *p<*0.01; Fig. 5B). In Bonferroni corrected one-tailed tests musical experience significantly correlated with NCI_SUB_ (*r*_s(17)_=0.63, *p<*0.01), but not NCI_RAW_ (*r*_s(17)_=−0.26, *p=*0.14) nor NCI_ADD_ (*r*_s(17)_=−0.13, *p=*0.30). However, as for the consonance preference measure, the correlation between musical experience and NCI_RAW_ became stronger when controlling for the effect of variation in NCI_ADD_, this time becoming significant (*r*_s(16)_=0.45, *p=*0.03). These results indicate that experience of playing a musical instrument is strongly associated with enhanced harmonicity of consonant dyads relative to dissonant dyads in the phase locking to TFS. When controlling for the effect of music experience using partial correlation, NCI_SUB_ was not found to correlate significantly with consonance preference (*r*_s(16)_=0.23, *p=*0.18), suggesting that the relation between representation of harmonicity in the FFR and consonance preference is driven by a co-dependence of each of these variables on musical experience.

Fig. 5C displays the relation between musical experience and the representation of harmonicity in FFR_SUB_ to dissonant and consonant dyads. Musical experience is only weakly associated with harmonicity of dissonant dyads (*r*_s_=0.28, *p*=0.25) but is strongly associated with harmonicity of consonant dyads (*r*_s_=0.52, *p*=0.02). The strong correlation between musical experience and NCI_SUB_ (black circles, solid line) is driven by an enhancement of the harmonicity of consonant dyads.

### 3.2 Behavioural ratings for diotic and dichotic stimuli

A two-way repeated-measures ANOVA with interval and presentation condition (diotic or dichotic) as factors confirmed a main effect of musical interval on pleasantness rating (*F*_(5, 135)_=210.60, *p<*0.01). There was also a significant effect of presentation condition on pleasantness rating (*F*_(1, 27)_=16.50, *p<*0.01) and a significant interaction between presentation condition and interval (*F*_(5, 135)_=54.60, *p<*0.01). In Bonferroni corrected pair-wise comparisons (*t*-tests), all consonant intervals were rated as being more pleasant in the diotic condition than in the dichotic condition (for the Perfect 4th, *t*_(27)_=8.60, *p<*0.01; for the Perfect 5th, *t*_(27)_*=*8.80, *p<*0.01; and for the Major 6th, *t*_(27)_=4.60, *p<*0.01). Of the dissonant intervals, only the Minor 2nd was more highly rated in the dichotic condition than in the diotic condition (*t*_(27)_=5.10, *p<*0.01). In other words, the interaction between presentation condition and interval was mainly driven by the fact that the consonant intervals tended to be more highly rated in the diotic condition. Importantly, the effect of consonant intervals being on average rated as more pleasant in the diotic condition (see Fig. 4A) meant that consonance preference scores calculated from pleasantness ratings of diotic stimuli were greater than consonance preference scores calculated from ratings of dichotic stimuli (*t*_(27)_=4.10, *p<*0.01).

### 3.3 Harmonic salience of the FFR for diotic and dichotic stimuli

A two-way repeated-measures ANOVA of harmonic salience in the FFR_RAW_ with interval and presentation condition as factors found a significant main effect of interval (*F*_(5, 90)_=65.35, *p*<0.01) which interacted with presentation condition (*F*_(5, 90)_=12.08, *p*<0.01). Presentation condition was not a significant main effect. To explore the interaction between interval and presentation condition further, Bonferroni corrected pair-wise comparisons of each interval were performed between diotic and dichotic conditions. The Perfect 5th (*t*_(18)_=3.19, *p*=0.05) had greater harmonic salience in the diotic condition and the Major 7th (*t*_(18)_=−5.92, *p*<0.01) had greater harmonic salience in the dichotic condition. Differences between presentation conditions were not significant for other intervals.

As can be seen in Fig. 4E, for FFR_RAW_ the difference between the mean harmonic salience of consonant and dissonant intervals was greater for diotic presentation than it was for dichotic presentation. Accordingly, the mean NCI score calculated for the diotic condition was significantly greater than the mean NCI score calculated for the dichotic condition (*t*_(18)_=3.47, *p=*0.03), indicating that the difference between harmonic salience in the FFR between consonant and dissonant intervals was greater in the diotic condition than in the dichotic condition.

**Fig. 6:**
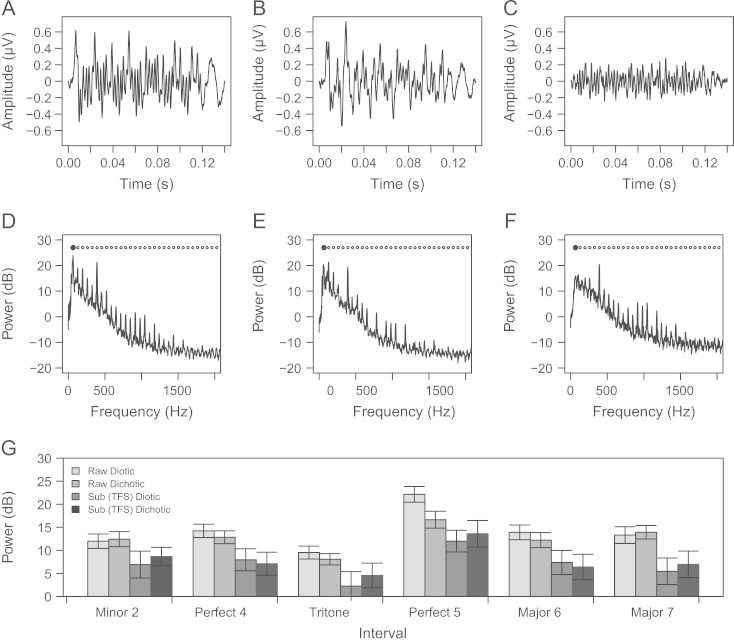
Waveforms (top) and spectra (middle) of the FFR to the Perfect 5th. Spectra are in dB referenced to 10^−16^ volts. The circles in the spectra (D–F) indicate a harmonic series with an *F*0 of 65.40 Hz. The figure displays the waveform (A) and spectrum (D) of the average FFR_RAW_ in the diotic condition, the waveform and (B) spectrum (E) of the average FFR_RAW_ in the dichotic condition, and the waveform (C) and spectrum (F) of the average FFR_SUB_ in the diotic condition. The bottom panel (G) displays the magnitude of the difference tone generated by interactions between the *F*0 of the root note and the interval note for each interval and FFR type. Error bars indicate 95% confidence intervals.

Fig. 6 displays the waveform of the diotic FFR_RAW_ (A), the dichotic FFR_RAW_ (B), and the diotic FFR_SUB_ (C) to the Perfect 5th dyad. The corresponding spectra are displayed in the middle row (Fig. 6D–F) in which harmonic series with an *F*0 of approximately 65 Hz are identifiable.^1^ The harmonics of the root note (*F*0=130.81 Hz) and the interval note (*F*0=196.00 Hz) are frequently separated by approximately 65 Hz, and as can be seen the spectrum of FFR_RAW_ for the diotic condition (Fig. 6D) contains a peak at 65 Hz that is much larger than in the dichotic condition (Fig. 6E), as confirmed by a Wilcoxon Signed Rank Test (*V*=177.00, *p*<0.01 *r*=−0.86). Note also the strong representation of the period corresponding to this frequency (approximately 15 ms) in the FFR waveform for the diotic condition (Fig. 6A). Likewise, the diotic FFR_RAW_ spectra to the other consonant intervals also contained significantly larger frequency components than the dichotic spectra at the difference tone between the F0s of the root and the interval note (Wilcoxon Signed Rank Tests, the Perfect 4th, *V*=145.00, *p*=0.02, *r=*−0.52, the Major 6th, *V*=159.00, *p*<0.01, *r*=−0.66), which in these cases correspond to the F0s of the best fitting harmonic series indicated in Table 1. Difference tones were not significantly different in magnitude between diotic and dichotic presentation for other intervals (see Fig. 6G).

The peaks at approximately 325 and 715 Hz (the 5th and 11th harmonics of a 65 Hz *F*0) in the spectrum for the diotic FFR_RAW_ (Fig. 6D) are also not present in the stimulus (see Fig. 3) nor the dichotic FFR_RAW_ spectrum (Fig. 6E). These presumably also result from interactions between frequency components in the two notes.

As can be seen in Fig. 6C, the suppression of the cochlear envelope component resulted in the FFR_SUB_ Perfect 5th waveform being lower in amplitude than the FFR_RAW_ waveforms. The large frequency component corresponding to the envelope component that is seen in the FFR_RAW_ spectra is significantly reduced in the FFR_SUB_ spectra (Wilcoxon Signed Rank Tests, Perfect 5th, *V*=188.00, *p*<0.01, *r*=−1.04, Perfect 4th, *V*=178.00, *p*<0.01, *r*=−0.80, Major 6th, *V=*190.00, *p*<0.01, *r*=−1.09; Fig. 6F).

To assess whether the enhanced NCI in the diotic condition was due to frequencies carried in the envelope produced by interactions on the basilar membrane, the effect of reducing the contribution of phase locking to the envelope to the FFR was explored by comparing the harmonic salience scores for consonant intervals relative to dissonant intervals for FFR_RAW_ and FFR_SUB_. A two-way repeated measures ANOVA of NCI with presentation condition and FFR type as factors found FFR type to be a main effect (*F*_(1,18)_=30.83, *p*<0.01) and that the two factors significantly interacted (*F*_(1, 18)_=47.27, *p*<0.01), indicating that the effect of presentation condition on the NCI depended upon whether contributions from the response to the envelope were included. Consonant intervals had greater harmonicity in the FFR relative to dissonant intervals when the components of the FFR to the envelope were included in the response (diotic FFR_RAW_).

For FFR_SUB_, the difference between the mean harmonic salience of consonant and dissonant dyads was greater for the dichotic presentation (Fig. 4E). This is discussed further in Section 4.3.

## 4 Discussion

### 4.1 Individual differences in consonance preference are related to individual differences in neural temporal coding

Large variation in fidelity of phase locking at the level of the brainstem as measured by the FFR has been shown to occur amongst even young and normal-hearing listeners previously (Ruggles, Bharadwaj, & Shinn-Cunningham, 2012; Ruggles & Shinn-Cunningham, 2011). Previous studies using the FFR have demonstrated a correlation between individual strength of phase locking and individual performance in tasks associated with pitch perception. Marmel et al. (2013) found strength of phase locking to correlate with performance in frequency discrimination even after variation explained by age and hearing loss had been partialed out. Similar correlations have also been demonstrated for F0 discrimination tasks (Bidelman et al. 2011a; Krishnan et al. 2010, 2012). In the present study, individual consonance preference significantly correlated with NCI_SUB._ The NCI is a measure of the salience of harmonicity in brainstem phase locking to consonant dyads relative to dissonant dyads. NCI_SUB_ is calculated from phase locking mainly to TFS. These results suggest that, despite envelope components driving an increase in both harmonicity of neural coding and consonance preference in diotic presentation when compared to dichotic presentation (see Section 4.3), individual variation in the acuity of neural coding of harmonically relevant TFS (i.e. individual harmonics) differentiates individual consonance preference.

The study also demonstrates a relation between individual musical experience and both individual behavioural and physiological measures of consonance. McDermott et al (2010) demonstrated a correlation between musical experience and both harmonicity preference and consonance preference. Previous studies have demonstrated effects of musical training on enhanced pitch tracking in the FFR (Bidelman et al. 2011b; Wong et al. 2007) and representation of musically relevant features of the spectrum of the FFR addition waveform (Lee, Skoe, Kraus, & Ashley, 2009). The results reported here suggest that musical experience results in enhanced phase locking to TFS but not to temporal envelope.

### 4.2 Diotic presentation results in both stronger consonance preference and stronger neural representation of harmonicity for consonant intervals

The results here demonstrate that ear presentation condition (diotic or dichotic) impacts upon the perceived pleasantness of consonant intervals, with consonant intervals being perceived as more pleasant when they are presented diotically (both notes to both ears) than when they are presented dichotically (each note to a different ear). The increased pleasantness of consonant intervals in the diotic condition results in a stronger overall preference for consonant dyads over dissonant dyads. To the authors׳ knowledge this is the first time that this effect has been demonstrated.

Previous work has provided evidence for a relation between the perception of consonance and pitch-relevant temporal information at the level of the human brainstem (Bidelman & Heinz, 2011; Bidelman & Krishnan, 2009, 2011). This earlier work measured the integrity of phase locking to the stimulus by examining the period corresponding to the *F*0 of the root note. Here it is demonstrated that each dyad׳s relative consonance is accounted for by the relative strength of harmonicity in the FFR. Moreover, the effect of presentation condition on harmonicity is likely the result of an enhancement of the harmonicity of consonant dyads via the addition of harmonically relevant components due to interactions in the cochlea (see Section 4.3). The present study builds upon previous behavioural work demonstrating that harmonicity is the driver of the perception of consonance (McDermott et al., 2010) by providing evidence that phase locking to the frequency components of the combined spectrum of a musical dyad is the physiological mechanism by which harmonicity is encoded in the auditory periphery.

### 4.3 Harmonicity of the FFR is enhanced in the diotic condition due to the addition of components produced by cochlear interactions

The results of the present study indicate that, whilst individual variation in phase locking to harmonically relevant TFS differentiates individual preference for consonance, phase locking to the amplitude modulation of the basilar membrane response drives a general preference for diotic over dichotic musical dyads. In the case of consonant intervals, the frequency of the amplitude modulation (the temporal envelope) corresponds to the best fitting *F*0 of the combined harmonics of the two notes of the dyad.

Tramo et al (2001) used autocorrelation to demonstrate that, for consonant dyads, the dominant period in the stimulus was also represented in the pooled all-order ISIs from 100 cat auditory nerve fibres. The authors suggested that phase locking in the auditory nerve could therefore be a mechanism for extracting this frequency from consonant musical stimuli. In the present study it was hypothesised that this frequency component would be greater in magnitude in the FFR to diotic dyads compared to dichotic dyads, due to cochlear temporal interactions between the harmonics of the two notes producing additional frequency components corresponding to the best fitting *F*0 (the sub-harmonic identified by Tramo et al). The results of this study provide evidence supporting this hypothesis. The *F*0 component representing the harmonic series of the combined spectrum of the two notes of consonant dyads is dominant in the diotic FFR_RAW_. That these frequency components are significantly reduced in the dichotic FFR_RAW_ suggests that they arise mainly from monaural processing, most likely interactions on the basilar membrane.

Attempts have been made to estimate the power of *propagated* cochlear distortion products previously, with evidence that the FFR to complex stimuli contains frequency components much larger than would be expected were they to have been generated in this way (Gockel, Farooq, Muhammed, Plack & Carlyon, 2012). Hence it is likely that the components corresponding to envelope arise from quadratic distortion in the transduction of the interacting harmonics, coded in the output of high frequency channels (Dau, 2003; Geisler, Rhode, & Kennedy, 1974; Kiang & Moxon, 1974). To test the hypothesis that additional frequency components in the FFR in the present study represent the envelope produced by the interaction of the two notes in the cochlea, the spectra of the FFR containing phase locking to the envelope (FFR_RAW_) and of the FFR with phase locking to the envelope suppressed (FFR_SUB_) were compared. It was found that these components were not present in the diotic FFR_SUB_, suggesting that this frequency component was produced by phase locking to the envelope of the cochlear response.

Phase locking to TFS is likely to be the primary mechanism for the coding of pitch (see Plack & Oxenham, 2005 for review). However, Moore and Moore (2003) demonstrated that the pitch of complexes consisting of only unresolved harmonics is likely to be determined by envelope rate: when the spectral envelope (and therefore basilar membrane excitation pattern) was held constant for complexes in which the harmonics were all shifted upwards in frequency by the same amount (therefore retaining the same envelope rate), the perceived pitch of complexes containing resolved harmonics shifted in proportion to the shift in frequency. However the pitch of complexes containing only unresolved complexes remained the same, suggesting that for these complexes pitch corresponded to the unchanged envelope rate. Houtsma and Smurzynski (1990) demonstrated that performance in frequency discrimination and pitch identification tasks was better for complexes containing resolved harmonics than for complexes containing only unresolved harmonics. However, performance when using complexes containing only unresolved harmonics improved with increasing number of harmonics present. This presumably demonstrates that the representation of the *F*0 in the envelope produced by interactions on the basilar membrane is enhanced with increasing number of unresolved harmonics. This could indicate the existence of two mechanisms of pitch perception: a primary pattern matching mechanism (e.g. Goldstein, 1973; Terhardt, 1979) dependant on access to TFS information; and a secondary mechanism for deriving pitch information from the envelope information produced by unresolved harmonics as suggested by Schouten (1940), as cited in Houtsma and Smurzynski (1990). The results of the current study suggest that this secondary mechanism could play an important role in ‘fusing’ the temporal information produced by the two notes of musical dyads. As suggested by Tramo et al (2001), the additional low frequency components present in the temporal coding of consonant dyads in the diotic condition correspond to the theoretical ‘fundamental bass’ note which Rameau (1971) believed to relate the individual notes of a consonant chord to one another. The present study demonstrates phase locking at a frequency corresponding to an *F*0 that defines the harmonicity of the chord and which serves to reinforce the fusion of the two notes into a single image in the manner suggested by Stumpf (1890). The findings of the present study suggest that envelope coding of cochlear interactions might serve as a mechanism for producing this sub-harmonic frequency component for notes not artificially separated by dichotic presentation.

The fact that an additional low-frequency component is identifiable at all in the *dichotic* FFR for consonant intervals (Fig. 6E) is noteworthy. A component corresponding to the difference tone between the *F*0s of the two notes is significantly larger in the FFR_RAW_ dichotic spectrum compared to the FFR_SUB_ diotic spectrum for the Perfect 4th (*V*=155.00, *p*=0.01, *r*=−0.62), Perfect 5th (*V*=155, *p*=0.01, *r*=−0.62) and Major 6th (*V*=166.00, *p*<0.01, *r*=−0.68). This suggests that the FFR may reflect interactions between harmonics at or after binaural integration in the superior olivary complex. Previous work has suggested that the FFR is not sensitive to such binaural interactions (Gockel, Carlyon, & Plack, 2011).

Interestingly, the results here demonstrate that when the response to the cochlear envelope is suppressed and the response to individual harmonics enhanced (FFR_SUB_), the harmonic salience of consonant dyads is significantly greater for dichotic dyads. Closer examination of spectra of the FFR_SUB_ to diotic and dichotic dyads reveals the absolute magnitude of the harmonic frequencies of the consonant dyads to be lower in the diotic case, therefore reducing the harmonic salience score. This may be an effect of the nonlinearity of the cochlea, made more extreme by the subtraction routine: where harmonics imperfectly coincide (due to the use of the equal temperament scale e.g. see equal temperament ratio to root note, Table 1) the response in the diotic case may be reduced compared to the dichotic case due to monaural neural suppression i.e. the neural synchrony of an auditory nerve fibre׳s response to a tone is suppressed by the addition of a second tone with a frequency slightly above or below the response region of the first tone (e.g. see Arthur, Pfeiffer & Suga, 1971). Where monaural suppression occurs it might be expected that frequencies in the FFR resulting from central binaural interactions to also be reduced in the diotic case.

### 4.4 Conclusions

Consonance preference for different musical intervals corresponded closely to the neural representation of harmonicity reflected in the FFR. Furthermore, individuals with a greater preference for consonance had a greater distinction between the representation of harmonicity in consonant and dissonant dyads in the FFR generated by phase locking to individual harmonics. When both notes of a consonant dyad were presented to both ears, the dyad was perceived as being more consonant than when the two notes were presented to separate ears. The FFR also revealed a stronger neural representation of harmonicity for consonant diotic dyads. When both notes were presented to both ears, interactions between the harmonics of the two notes on the basilar membrane resulted in additional frequency components being present in the FFR. These components enhanced the harmonicity of the FFR, suggesting that this could be the physiological mechanism for the increased perception of consonance in the diotic condition. Overall, the results suggest that consonance preference depends in part on the sub-cortical neural temporal representation of harmonics and their cochlear interactions.

## Figures and Tables

**Table 1:** 

**Interval (semitones)**	**Interval (name)**	**F0 (Hz)**	**Equal temperament ratio to root note**	**F0 of best fitting harmonic series (Hz)**
1	Minor 2nd	138.59	1.05946	46.20
5	Perfect 4th	174.61	1.33483	43.71
6	Tritone	185.00	1.41421	37.11
7	Perfect 5th	196.00	1.49831	32.70
9	Major 6th	220.00	1.68179	43.71
11	Major 7th	246.94	1.88775	43.58

## Footnotes

1 This frequency is a C note one octave below the root note, and corresponds to the second harmonic of a 32.70 Hz harmonic series. In the harmonic sieve analysis of the Perfect 5th stimulus, 65.40 Hz was marginally less salient than 32.70 Hz. However this is due to the greater number of bins in sieves with lower *F*0s. The 32.70 Hz series fits well because the harmonics coincide with those of the 65.40 Hz series.
